# Supplementary material for: Chromogranin A deficiency attenuates tauopathy by altering epinephrine–alpha-adrenergic receptor signaling in PS19 mice
Source: Nat Commun. 2025 May 20;16:4703. doi: 10.1038/s41467-025-59682-6 (PMC12092710; doi:10.1038/s41467-025-59682-6)
Supplement: Supplementary file 1 — Supplementary Information [file 41467_2025_59682_MOESM1_ESM.pdf]

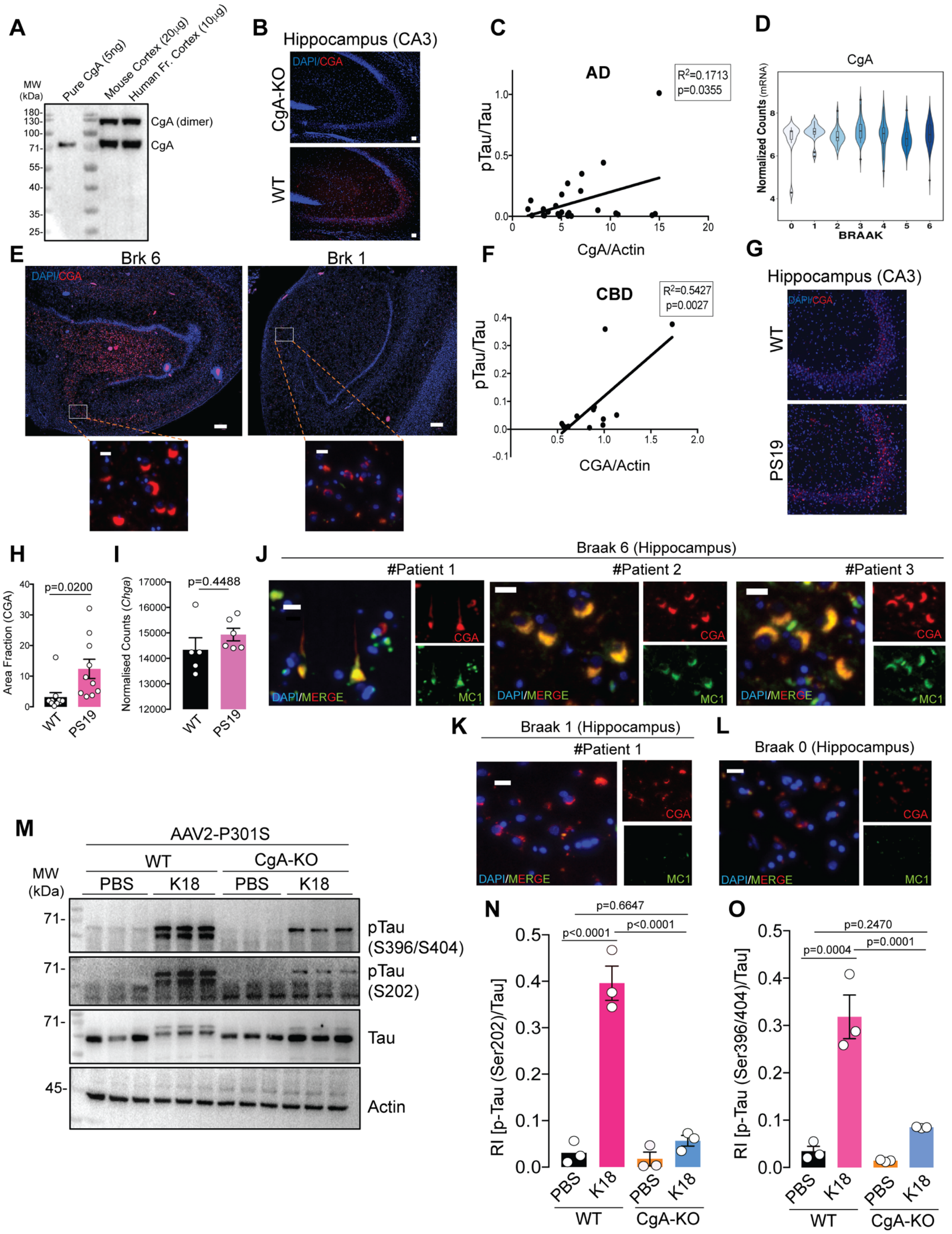

Supplementary Fig 1. CgA co-localize with aggregated Tau (MC1) and CgA depletion in OTSC reduces Tau seeding.

A. Immunoblotting with pure CgA and lysate from mouse and human brain.
B. IHC showing depletion of CgA in CgA-KO. Scale bar = 50 μm
C. Pearson correlation of pTau/Tau and CgA/Actin in AD.

D. Quantification of *CHGA* (gene encoding CgA) transcript levels of patient samples from all six Braak stages. The RNAseq data was obtained from publicly available AD-AMP dataset.

E. Low Magnification image of Braak6 and Braak1 hippocampi with high magnification image of specified location inset. Scale bar = 400 μm

F. Pearson correlation of pTau/Tau and CgA/Actin in CBD.

G-H. Representative image of IHC of CgA in WT and PS19 mice hippocampus (G) with quantification (H). Scale bar = 20 μm. P-value was calculated using Unpaired two-tailed T-test with Welch's correction, t = 2.658, df = 12.75.

I. Transcript level of CgA in WT (n=5) and PS19 mice (n=6) (t = 0.8106, df = 5.947)

J-K. Co-localization of CgA (red) and MC1 positive Tau filaments (green) in three different Braak 6 (J) and one Braak 1 (K) hippocampi. Scale bar = 20 μm.

L. Co-localization of CgA (red) and MC1 positive Tau (green) in Braak 0 hippocampi. Scale bar = 20 μm.

M. WB showing reduced pTau in AAV2-PS19 (P301S) transduced CgA-KO slices compared to WT.

N-O. Densitometric quantification of phospho-Tau (Ser396/404, Ser202) between K18-treated and AAV2-hTau (P301S) transduced CgA-KO (n = 3) and WT (n = 3) slices. P-value was calculated using One-way Anova (I; $F_{3, 8}$ = 0.6742, P = 0.5917, J; $F_{3, 8}$ = 1.547, P = 0.2759) Sidak's multiple comparison test. Data are presented as mean values +/- SEM. Source data are provided as a Source Data file.

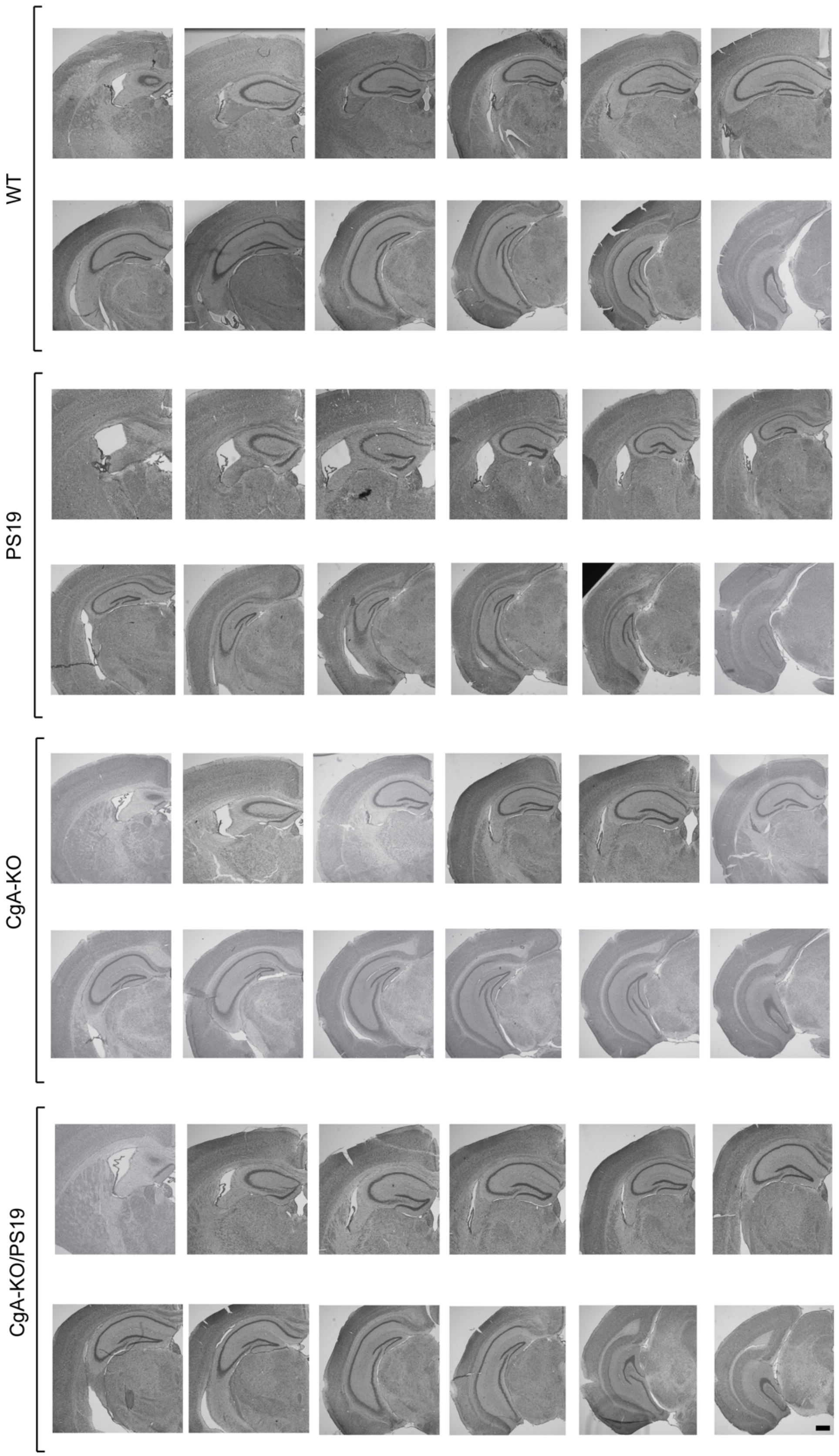

WT
PS19
CgA-KO
CgA-KO/PS19

Supplementary Fig 2. Series of hippocampus image for WT, PS19, CgA-KO and CgA-KO/PS19. Scale bar = 200 μm.

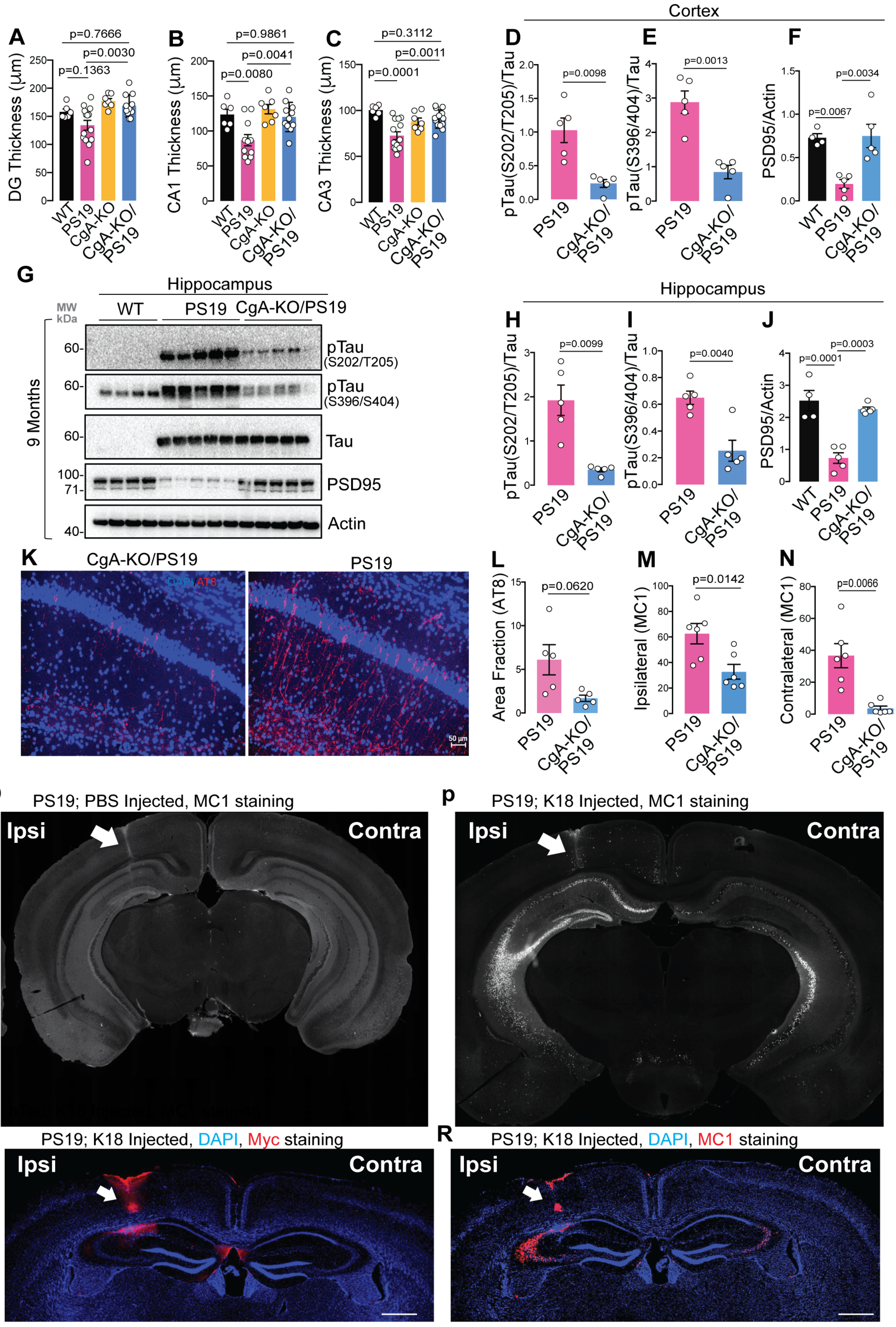

A
DG Thickness (µm)
p=0.7666
p=0.0030
p=0.1363
WT
PS19
CgA-KO
CgA-KO/PS19
B
CA1 Thickness (µm)
p=0.9861
p=0.0041
p=0.0080
C
CA3 Thickness (µm)
p=0.3112
p=0.0011
p=0.0001
Cortex
D
pTau(S202/T205)/Tau
p=0.0098
E
pTau(S396/404)/Tau
p=0.0013
F
PSD95/Actin
p=0.0034
p=0.0067
G
Hippocampus
MW kDa
WT
PS19
CgA-KO/PS19
9 Months
pTau (S202/T205)
pTau (S396/S404)
Tau
PSD95
Actin
Hippocampus
H
pTau(S202/T205)/Tau
p=0.0099
I
pTau(S396/404)/Tau
p=0.0040
J
PSD95/Actin
p=0.0003
p=0.0001
K
CgA-KO/PS19
PS19
DAPI/AT8
50 µm
L
Area Fraction (AT8)
p=0.0620
M
Ipsilateral (MC1)
p=0.0142
N
Contralateral (MC1)
p=0.0066
O
PS19; PBS Injected, MC1 staining
Ipsi
Contra
p
PS19; K18 Injected, MC1 staining
Ipsi
Contra
Q
PS19; K18 Injected, DAPI, Myc staining
Ipsi
Contra
R
PS19; K18 Injected, DAPI, MC1 staining
Ipsi
Contra

Supplementary Fig 3. Rescue of tau pathology in CgA-KO/PS19 mice.

A. Image J quantification of DG thickness in WT (n=6), PS19 (n = 14), CgA-KO/PS19 (n = 11) and CgA-KO mice (n = 7). P-values were calculated using One-way ANOVA ($F_{3, 34}$ = 6.807, p = 0.0010) Sidak's Multiple Comparison test.

B. Image J quantification of CA1 in WT (n = 6), PS19 (n = 11), CgA-KO/PS19 (n = 11), and CgA-KO (n = 7) mice. P-values were calculated using One-way ANOVA ($F_{3, 31}$ = 7.521, p = 0.0006) Sidak's Multiple Comparison test.

C. Image J quantification of CA3 thickness in WT (n=6), PS19 (n=14), CgA-KO/PS19 (n=13) and CgA-KO (n=7) mice. P-values were calculated using One-way ANOVA ($F_{3, 36}$ = 9.392, p = 0.0001) Sidak's Multiple Comparison test.

D-F. Densitometric quantification of WB of pTau (C & D) in CgA-KO/PS19 (n = 5) and PS19 (n = 5) mice, and PSD95 (E) in WT (n = 4), CgA-KO/PS19 (n = 5) and PS19 (n = 5) mice by image lab (n = 6). P-value for D and E was calculated using Unpaired two-tailed T-test with Welch's correction (D; t = 4.132, df = 4.827, E; t = 5.348, df = 6.545). P-value for F was calculated using One-way Anova ($F_{2, 11}$ = 11.84, P = 0.0018) Sidak's Multiple Comparison Test.

G. WB of hippocampus RIPA lysates of WT (n = 4), PS19 (n = 5) and CgA-KO/PS19 (n = 5) mice showing levels of pathogenic phosphorylated Tau (pTau), total Tau, PSD95 and actin.

H-J. Densitometric quantification of WB of pTau (C & D) in CgA-KO/PS19 (n = 5) and PS19 (n = 5) mice, and PSD95 (E) in WT (n = 4), CgA-KO/PS19 (n = 5) and PS19 (n = 5) mice by image lab (n = 6). P-value for D and E was calculated using Unpaired two-tailed T-test with Welch's correction (H; t = 4.523, df = 4.126, I; t = 4.26, df = 6.787). P-value for J was calculated using One-way Anova ($F_{2, 11}$ =26.25, P<0.0001) Sidak's Multiple Comparison Test.

K-L. Representative IHC images of p-Tau (Ser 202/Thr205) with quantification in CgA-KO/PS19 and PS19 mice (n = 5). In G, p-value was calculated using unpaired two-tailed T-test with Welch's correction, t = 2.498, df = 4.346.

M-N. Quantification of MC1+ Tau seeds in ipsilateral (H) and contralateral (I) side of the brain. P-values were calculated using unpaired two-tailed T-test with Welch's correction (H; t = 3.024, df = 9.095, I; t = 4.287, df = 5.395).

O. PBS injected PS19 showing no MC1 staining. White arrow marks the area of injection.

P. K18 injected PS19 showing MC1 staining. White arrow marks the area of injection.

Q. MyC staining showing K18 (Myc tagged) injection in the ipsilateral hippocampus and K18 is not spread to the contralateral hippocampus. White arrow marks the area of injection.

R. MC1 staining shows the spreading of Tau from ipsilateral to contralateral hippocampus of the same brain used in Q. White arrow marks the area of injection. Scale bar = 200 μm. Data are presented as mean values +/- SEM. Source data are provided as a Source Data file.

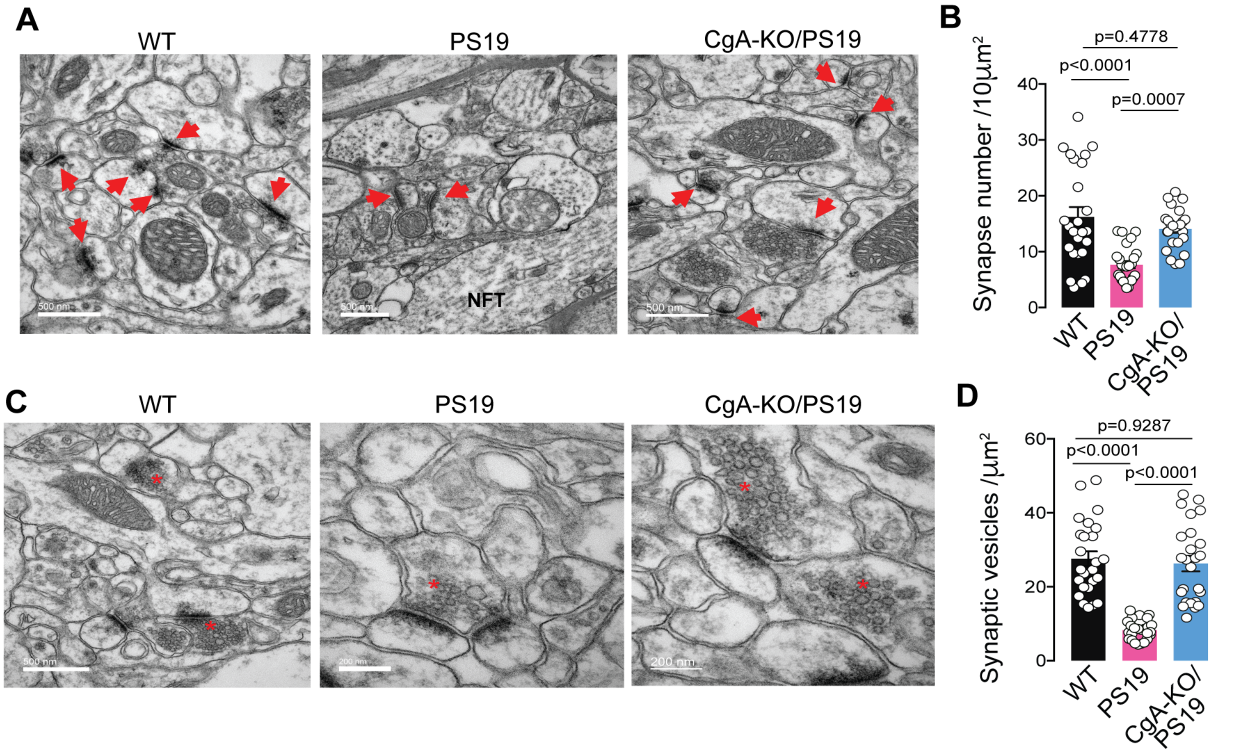

Supplementary Fig 4. Ultrastructure analysis of WT, PS19 and CgA-KO/PS19 brain.

A-B. Transmission electron microscopy (TEM) image showing synapse density in CgA-KO/PS19 (n=25; 25 micrographs from 3 animals), PS19 (n=25; 25 micrographs from 3 animals) and WT (n=25; 25 micrographs from 3 animals) mice, quantified in P as a function of synapse number measured in $10\mu m^2$. P-value in B was calculated using One-way ANOVA ($F_{2, 72} = 10.74$, P<0.0001) Tukey's Multiple Comparison test.

C-D. TEM images showing synaptic vesicles in CgA-KO/PS19 (n=25; 25 micrographs from 3 animals), PS19 (n=25; 25 micrographs from 3 animals) and WT (n=25; 25 micrographs from 3 animals) mice depicted by TEM images and quantification (D). P-values in D were calculated using One-way ANOVA ($F_{2, 72} = 13.95$, P<0.0001) Tukey's Multiple Comparison test. Data are presented as mean values +/- SEM. Source data are provided as a Source Data file.

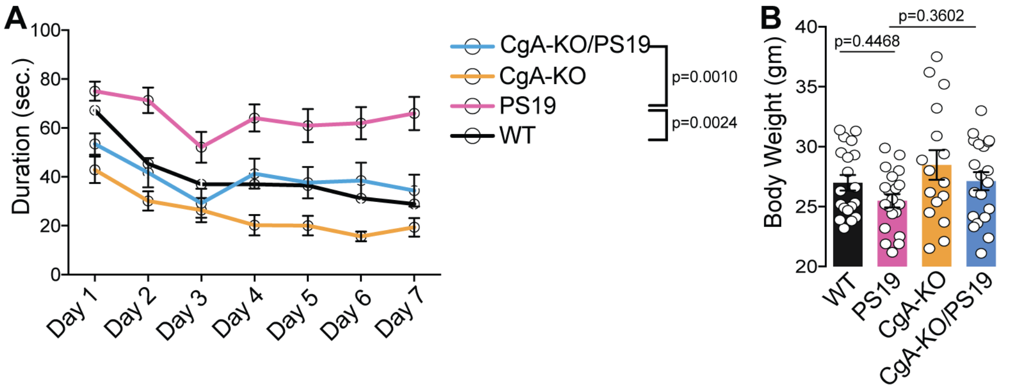

Supplementary Fig 5. Improved cognitive and motor function in CgA-KO/PS19 mice compared to PS19 mice.

A. Time taken to reach the platform in each day of MWM by all four mice group [WT (n = 18), CgA-KO (n = 16), PS19 (n = 19) and CgA-KO/PS19 (n = 20)]. P-value was calculated using Two-way ANOVA ($F_{18,\ 414}$ = 1.921, P = 0.0132) Turkey's Multiple comparison test.

B. Body weight for all four mice groups (WT, CgA-KO, PS19 and CgA-KO/PS19). [WT (n = 19), CgA-KO (n = 20), PS19 (n = 16) and CgA-KO/PS19 (n = 20). p-value was calculated using One-way ANOVA ($F_{3,\ 71}$ = 2.255, P=0.0894) Sidak's Multiple comparison test. Data are presented as mean values +/- SEM. Source data are provided as a Source Data file.

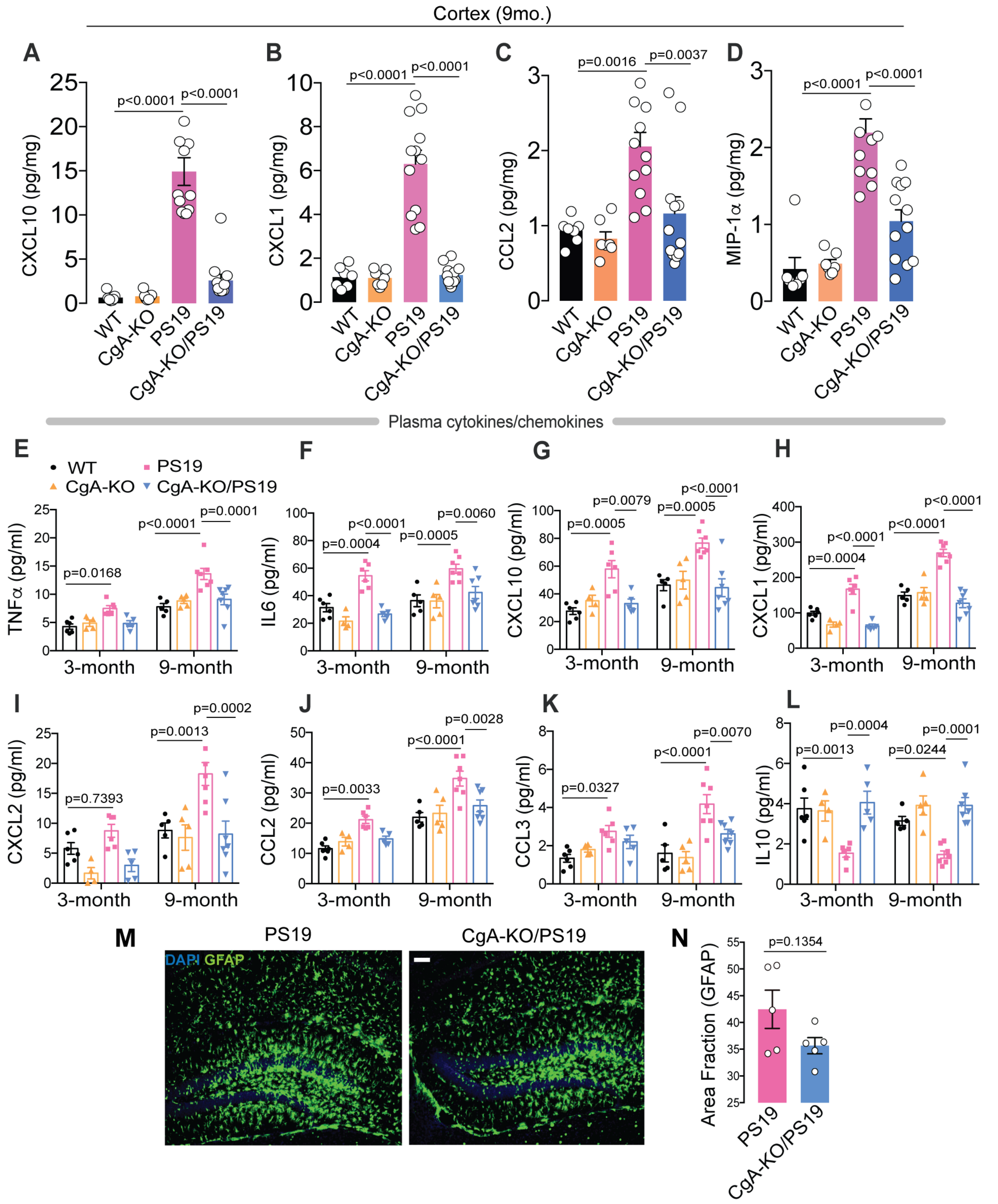

Supplementary Fig 6. CgA-KO/PS19 mice exhibit decreased microgliosis compared to PS19 mice

A-D. Inflammatory cytokine levels in the cortex of WT (n = 7), PS19 (n = 12), CgA-KO (n = 7) and CgA-KO/PS19 (n = 12) mice at 9 months of age (mo). P-values were calculated using One-way ANOVA Turkey's Multiple Comparison Test. A: CXCL10; $F_{3, 34} = 42.98$, P<0.0001, B: CXCL1; $F_{3, 34} = 43.82$, P<0.0001, C: CCL2; $F_{3, 34} = 9.272$, P = 0.0001, D: CCL3; $F_{3, 34} = 27.79$, P<0.0001.

E-L. Plasma inflammatory cytokine levels at 3 mo and 9 mo age in WT, PS19, CgA-KO and CgA-KO/PS19 mice. [3 mo; WT (n = 6), CgA-KO (n = 5), PS19 (n = 6), CgA-KO/PS19 (n = 5) and 9 mo; WT (n = 5), CgA-KO (n = 5), PS19 (n = 7), CgA-KO/PS19 (n = 7)]. P-values were calculated using Two-way ANOVA Sidak's multiple comparison test. E: TNFα; $F_{3, 37} = 1.317$, P = 0.2836, F: IL6; $F_{3, 37} = 1.171$, P = 0.3338, G: CXCL10; $F_{3, 37} = 0.2628$, P = 0.8517, H: CXCL1; $F_{3, 37} = 2.406$, P = 0.0828, I: CXCL2; $F_{3, 37} = 1.426$, P = 0.2507, J: CCL2; $F_{3, 37} = 0.4855$, P = 0.6944, K: CCL3; $F_{3, 37} = 0.4855$, P = 0.6944, L: IL10; $F_{3, 37} = 0.3789$, P = 0.7688.

M. Representative IF images showing the levels of GFAP in the hippocampi (CA1 region) of PS19 and CgA-KO/PS19 mice. Scale bar = 50 μm.

N. Quantification of GFAP fraction area calculated from IF mages as shown in G (n = 5 per group). P-value was calculated using Unpaired two-tailed T-test with Welch's correction, t = 1.754, df = 5.399. Data are presented as mean values +/- SEM. Source data are provided as a Source Data file.

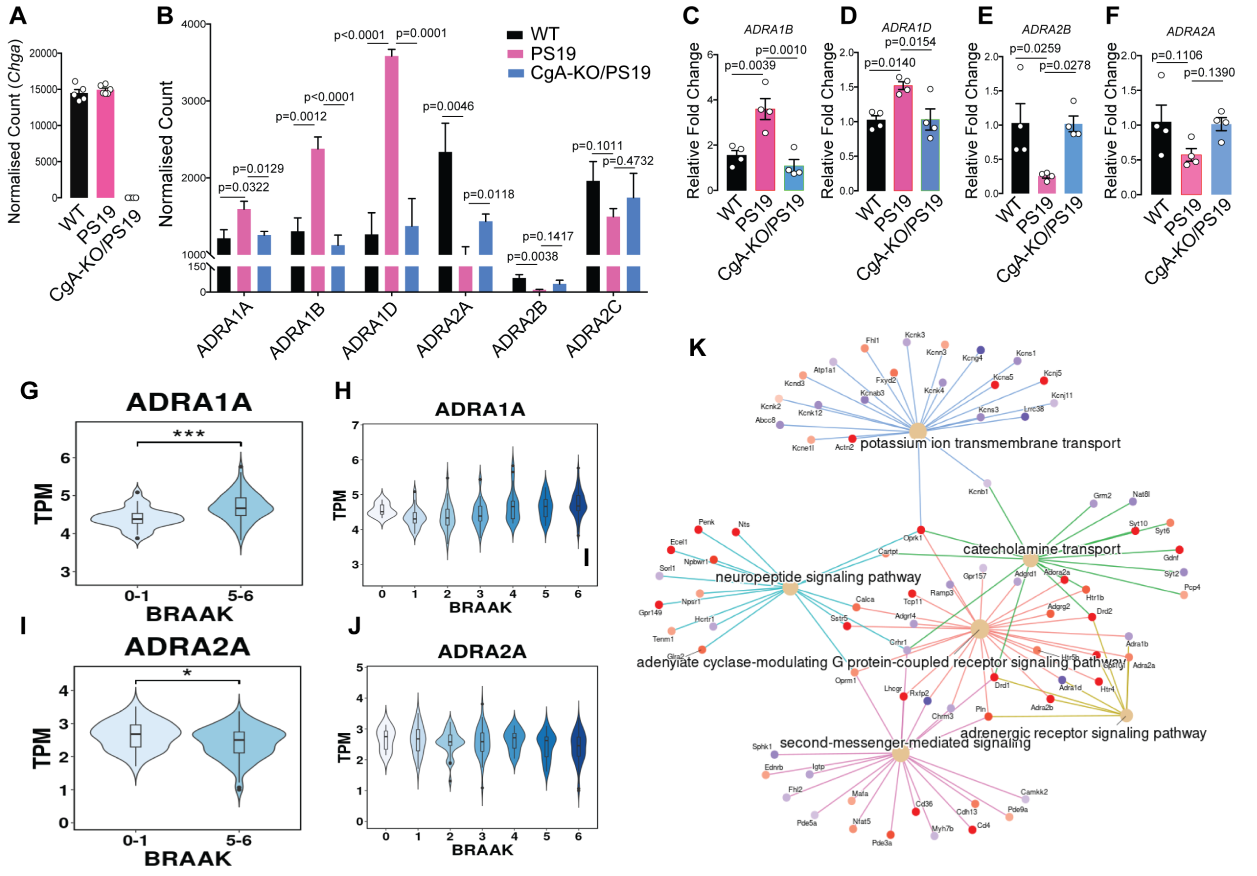

Supplementary Fig. 7. Validation of transcriptional changes of genes identified in RNA-seq of WT, PS19 and CgA-KO/PS19 hippocampus.

- A. Normalized count of Chga mRNA in WT, PS19 and CgA-KO/PS19.
- B. Normalized counts of class I and class II alpha-adrenergic receptors in WT, PS19 and CgA-KO/PS19 hippocampus. P-values were calculated using Two-way ANOVA Dunnett's multiple comparison test ($F_{10, 84}$ = 13.88, P<0.0001)
- C-F. qPCR of genes in WT, PS19 and CgA-KO/PS19. P-values were calculated using One-way ANOVA (C; $F_{2, 9}$ = 15.85, P = 0.0011, D; $F_{2, 9}$ = 7.907, P = 0.0104, E; $F_{2, 9}$ = 2.787, P = 0.1143, F; $F_{2, 9}$ = 6.255, P = 0.0198) Sidak's multiple comparison test.
- G-J. Analysis of alpha-1 adrenergic receptor transcript level in Braak stage 1 and Braak stage 6 patients (parahippocampal gyrus) from AMP-AD database.
- K. Network analysis of the pathway involved depicting adrenergic signaling pathway as an important one linked with catecholamine transport, neuropeptide signaling and potassium ion transmembrane transport. Data are presented as mean values +/- SEM. Source data are provided as a Source Data file.

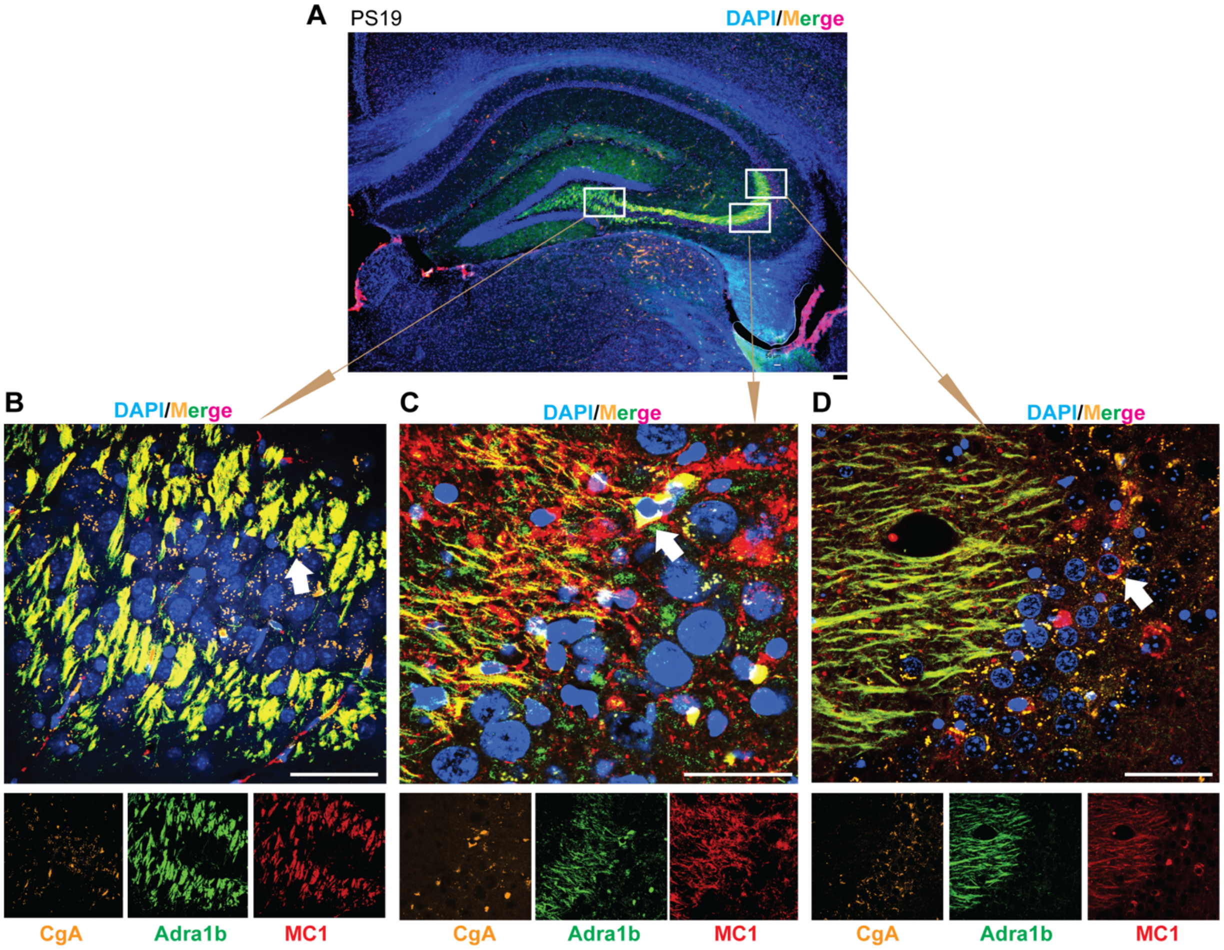

Supplementary Fig 8. Immunohistochemistry of CgA, Adra1b and Tau filaments in PS19 hippocampus.

A. Low magnification image of the PS19 hippocampus. Scale bar = 100 μm.

B. High magnification image of the CA4 region of the hippocampus. The region of hippocampus is showed by box in A. Scale bar = 50 μm.

C-D. High magnification image of CA3 region of hippocampus for CgA, Adra1b and Tau filaments. Region of hippocampus selected is encircled by white box in A. Scale bar = 50 μm.

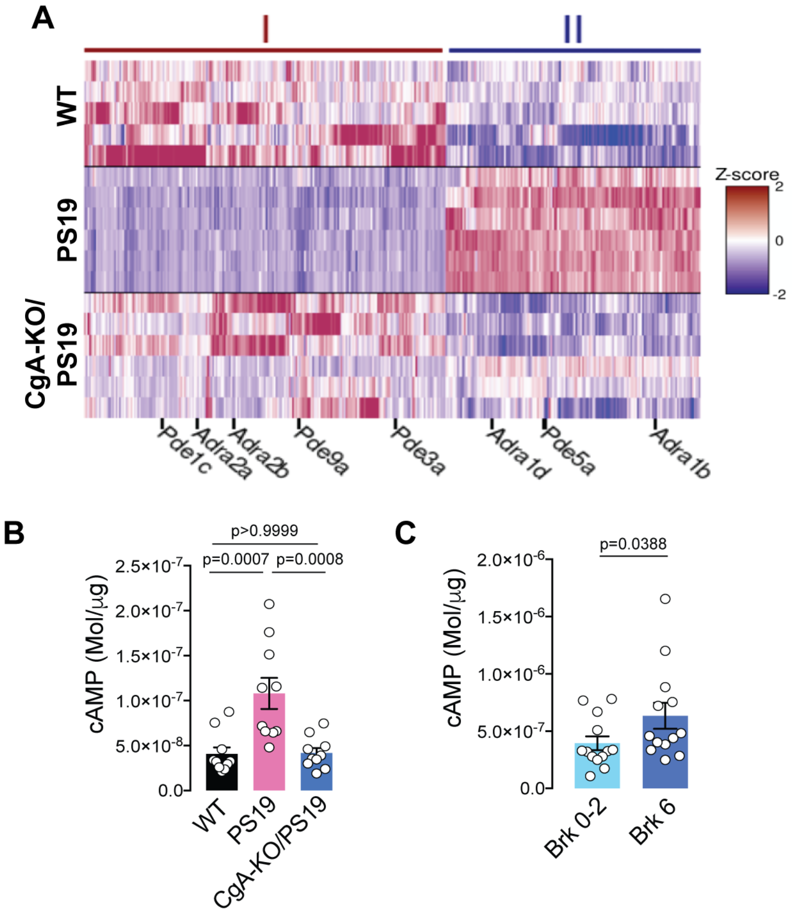

Supplementary Fig. 9. Enhanced cAMP and Phosphodiesterase (PDE) level in CgA-KO/PS19 mice.

A. RNA-seq analysis of differentially expressed genes (DEGs) showing the expression of different phosphodiesterases (PDEs) across the three genotypes (WT, PS19 and CgA-KO/PS19).
B. Decreased cAMP level in the cortex of CgA-KO/PS19 mice compared to PS19 mice. N = 10. P-values were calculated using One-way ANOVA ($F_{2, 27} = 11.75$, $P = 0.0002$) Sidak's Multiple comparison test.
C. Increased cAMP level in Braak stage 6 patient (n=13) cortex lysates compared to Braak stage 1-2 (n=13). P-value is calculated using Unpaired Two-tailed T-test, t = 1.8666, df = 24. Data are presented as mean values +/- SEM. Source data are provided as a Source Data file.

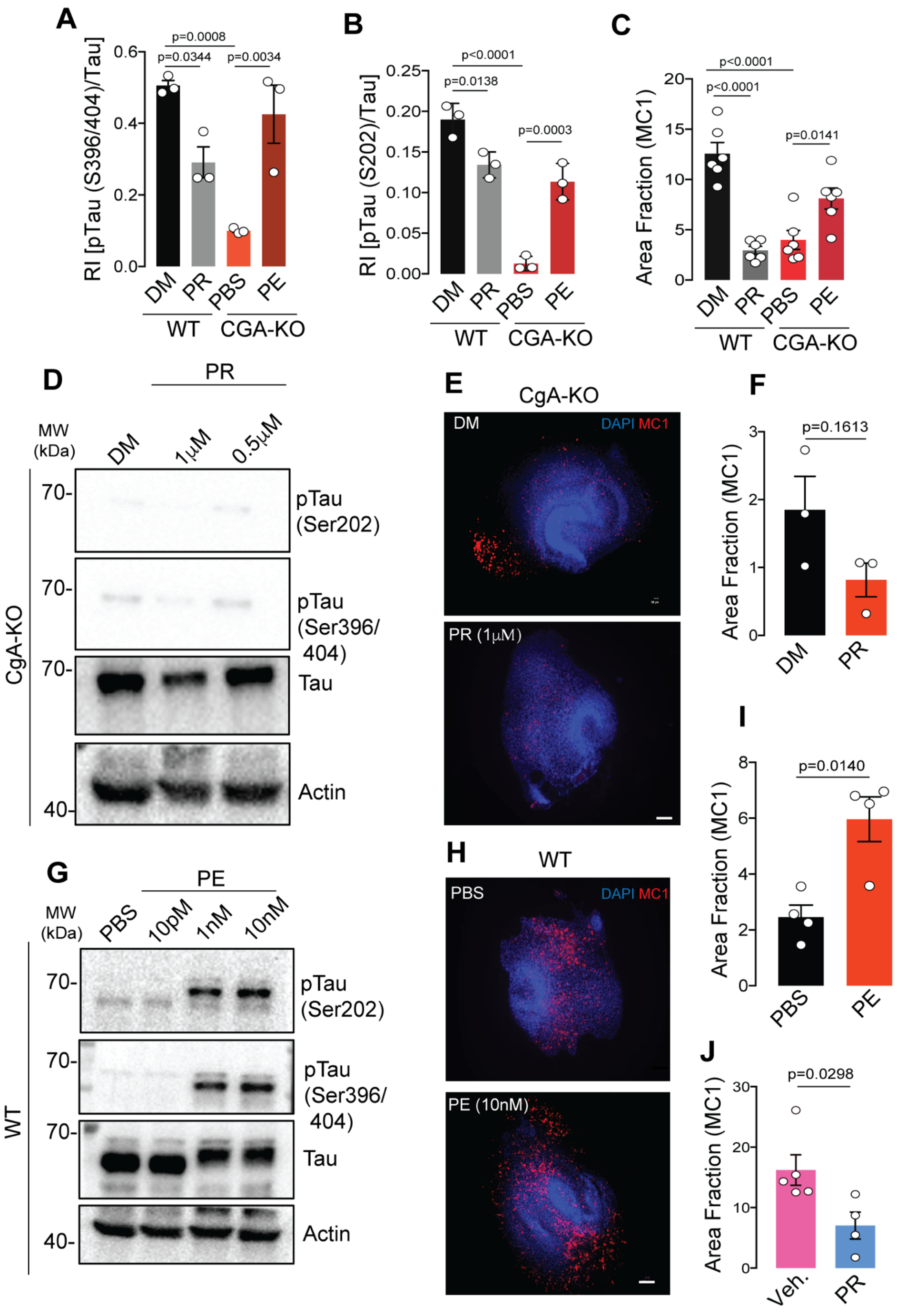

Supplementary Fig 10. Alpha-1 Adrenergic receptor regulates Tau phosphorylation and formation of neurofibrillary tangles.

A. WB quantification of Tau phosphorylation at S396/S404 relative to total tau levels (n=3). P-value was calculated using One-way Anova ($F_{3, 8}$ = 0.6682, P = 0.0013) Sidak's multiple comparison test.
B. WB quantification of Tau phosphorylation at S202 relative to total tau levels (n=3). P-value was calculated using One-way Anova ($F_{3, 8}$ = 0.3892, P<0.0001) Sidak's multiple comparison test.
C. Image J quantification of MC1+ misfolded Tau species in OTSC as percentage of total slice area from IF images as shown in E. (n = 6 per group). P-value was calculated using One-way Anova ($F_{3, 20}$ = 0.5465, P<0.0001) Sidak's multiple comparison test.
D. Western blot showing pTau in Prazosin (PR) treated CgA-KO OTSC.
E. Representative IHC image of DMSO (DM) and PR treated CgA-KO OTSC. Scale bar = 200 μm.
F. Image J quantification of the image in E (t = 1.861 df = 2.948).
G. Western blot showing pTau in Phenylephrine (PE) treated WT OTSC
H. Representative IHC image of PBS and PE treated WT OTSC.
I. Image J quantification of the image in G (t = 3.849, df = 4.619)
J. ImageJ quantification of MC1 staining in veh. (n = 5) And PR (n = 4) treated PS19 mice. P-value was calculated using unpaired two-tailed T-test with Welch's correction (t = 2.72, df = 6.998). Data are presented as mean values +/- SEM. Source data are provided as a Source Data file.

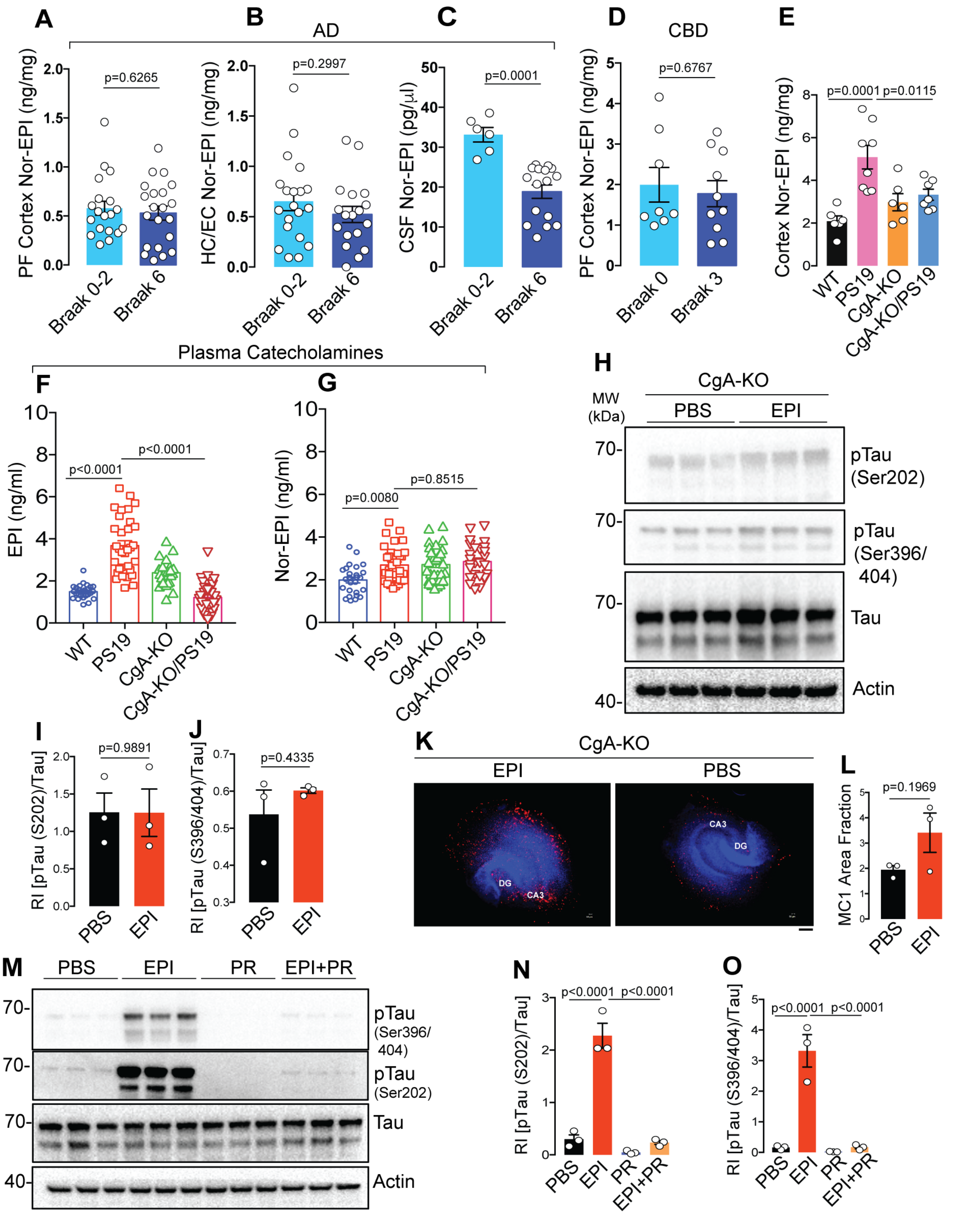

Supplementary Fig 11. Catecholamine levels in AD and CBD patient cortex and in CgA-KO/PS19 mice plasma.

A-C. Norepinephrine (NE) levels in Braak 6 and Braak 0-2 AD prefrontal cortex (A), hippocampus/entorhinal cortex (B) and CSF (C). cortex [Braak 0-2 (n = 19), Braak 6 (n = 21)], hippocampus [Braak 0-2 (n = 18), Braak 6 (n = 20)]. P-values were calculated using unpaired two-tailed T-test. (A; t = 4.907, df = 38, B; t = 1.052, df = 36, C; t = 4.812, df = 20)

D. NE levels in CBD pre-frontal cortex. Braak 0 (n = 8), Braak 3 (n = 10). P-values were calculated using unpaired two-tailed T-test, t = 0.4247, df = 16.

E. NE levels in cortex of WT (n = 6), PS19 (n = 7), CgA-KO (n = 6) and CgA-KO/PS19 (n = 7) mice. P-values were calculated using One-way ANOVA, ($F_{3, 23}$ = 9.88, P = 0.0002) Dunnett's multiple comparison test.

F. EPI level in plasma of WT (n = 26), PS19 (n = 33), CgA-KO (n = 26) and CgA-KO/PS19 (n = 32) mice. P-values were calculated using One-way ANOVA ($F_{3, 108}$ = 12.08, P<0.0001) Sidak's multiple comparison test.

G. NE levels in plasma of WT (n = 26), PS19 (n = 33), CgA-KO (n = 26) and CgA-KO/PS19 (n = 32) mice. P-values were calculated using One-way ANOVA ($F_{3, 109}$ = 4.348, P=0.0062) Sidak's multiple comparison test.

H. Western blot of pTau in PBS and Epinephrine (EPI) treated CgA-KO OTSC.

I-J. Densitometric quantification of pTau in PBS (n = 3) and EPI (n = 3) treated CgA-KO slice (I; t = 0.01463, df = 3.843. J; t = 0.9668, df = 2.05)

K. Representative IHC image of MC1 staining in PBS and EPI treated CgA-KO OTSC. Scale bar = 200 μm.

L. ImageJ quantification of MC1 area fraction in PBS (n = 3) and EPI (n = 3) treated CgA-KO OTSC (t=1.839, df = 2.175).

M. Western blot showing pTau in PBS, Epinephrine (EPI), Prazosin (PR) and EPI+PR treated WT OTSC.

N-O. Densitometric quantification of pTau in PBS (n = 3), EPI (n = 3), PR (n = 3) and EPI+PR (n = 3). P-value was calculated using One-way Anova (N; $F_{3, 8}$ = 66.65, P<0.0001, O; $F_{3, 8}$ = 36.26, P<0.0001) Sidak's Multiple Comparison Test. Data are presented as mean values +/- SEM. Source data are provided as a Source Data file.

Supplementary Table 1:
Alzheimer's Disease Patient sample details:

PATHID	REGTRYID	CSFID	PATHDX1	BRAAK1	SEX
5919	3454	12/064	Normal	1	F
5909	3194	5909	Primary Age-Related Tauopathy (PART)	2	M
5903	8176	8176-12	Primary Age-Related Tauopathy (PART)	2	M
5864	8011	5864	Primary Age-Related Tauopathy (PART)	2	M
5844	2115	5844	Normal	2	F
5687	3396	12/032	Normal	1	M
5856	3354		Alzheimer's changes	2	M
5747	3046		Alzheimer's changes	2	M
5699	14043		Alzheimer's changes	2	F
5662	14005		Alzheimer's changes	2	F
5655	2785		Alzheimer's changes	2	F
5601	8132		Alzheimer's changes	2	M
5510	2621		Alzheimer's changes	1	M
5495	2853		Alzheimer's changes	2	M
5447	14080		Alzheimer's changes	1	F
5434	2894		Alzheimer's changes	2	M
5398	14083		Alzheimer's changes	0	F
5381	2963		Alzheimer's changes	2	M
5372			Normal	1	F
5356	2861		Alzheimer's changes	1	F
**Chen 22-25 CSF + fHip/EC + fFC**					
PATHID	REGTRYID	CSFID	PATHDX1	BRAAK1	SEX
5945	3448	15/031	Alzheimer's disease	6	M
5937	3183	5937	Alzheimer's disease	6	F
5933	8052	5933	Alzheimer's disease	6	F
5930	3070	5930	Alzheimer's disease	6	F
5928	3265	13/010	Alzheimer's disease	6	F
5921	3435	13/057	Alzheimer's disease	6	F
5912	3188	13/002	Alzheimer's disease	6	F
5906	3532	11/014	Alzheimer's disease	6	F
5899	3682	5899	Alzheimer's disease	6	F
5892	3449	5892	Alzheimer's disease	6	F
5889	3213	5889	Alzheimer's disease	6	F
5885	3845	12/013	Alzheimer's disease	6	F
5838	3065	5838	Alzheimer's disease	6	M
5834		15/027	Alzheimer's disease	6	F
5832	3569	13/024	Alzheimer's disease	6	M

5830	3523	15/004	Alzheimer’s disease	6	F
5931	3088		Alzheimer’s disease	6	F
5918	8233		Alzheimer’s disease	6	F
5914	19008		Alzheimer’s disease	6	M
5905			Alzheimer’s disease	6	M

Supplementary Table 2:
Corticobasal Degeneration (CBD) patient sample details:

ID	Disease	Braak stage	Clinical ID	Family history	SEX
bb20009	CBD/PART/AGD/ARTAG/SRT	3	CBD	Yes	M
bb20061	CBD/AGD/ARTAG/PART	3	CBD	No	M
bb20082	CBD/AGD/ARTAG/PART	3	FTD	No	M
bb20171	CBD/AGD/TSA (ARTAG)/SC/Binswanger	3	FTD	Yes	F
bb20225	CBD/SC/ARTAG	3	CBD	No	M
bb20299	CBD/PSD(PNLA)/AGD/ARTAG/SC	3	CBD	No	M
bb20335	CBD/AGD/ARTAG/PART	3	CBD	No	F
bb21098	CBD/PART/ARTAG	3	FTD	No	F
bb21201	CBD (CBD-Cog)/CAA/PART	3	CBD	No	M
bb20005	PSP/ARTAG	0	PSP	No	M
bb20311	PSP (pure)	0	PSP	No	M
bb20380	PSP (cbl ctx & dentate)/SC	0	CBD	Yes	F
bb21419	CBD (PNLA)/SC/AGD/ARTAG	0	CBD	No	F
bb21004	Normal (pseudo-dementia)	0	CBD	No	M
bb21204	Normal (pseudo-dementia, drug-related parkinsonism)/AGD (mild)/ARTAG	0	depression (ECT/VNS)/PD	No	M
bb21537	Normal	0	All	No	F
bb23173	Normal (GAD)	0	Anxiety Disorder	No	M
bb22281	PART/brainstem encephalitis/depressive pseudodementia	0	FTD	No	F

Supplementary Table 3: qPCR Forward and Reverse Primer sequence details:

Gene name	Forward	Reverse
ADRA1B	5' GAAAGCAGCCAAAACCTTGGGC 3'	5' CACTACCTTGAATACGGCGTCC 3'
ADRA1D	5' GTGTCTTCGTCCTGTGCTGGTT 3'	5' GCCAGAAGATGACCTTGAAGACG 3'
ADRA2A	5' CAGGTGACACTGACGCTGGTTT 3'	5' GACACCAGGAAGAGGTTTTGGG 3'
ADRA2B	5' GTGTGCCATCAGTCTGGACAGA 3'	5' CAATGAGCCACACGGTGAGGAT 3'
β-ACTIN	5' CATTGCTGACAGGATGCAGAAGG 3'	5' TGCTGGAAGGTGGACAGTGAGG 3'

Suplementary Table 4: Summary of the RNA-Seq data derived from AMP-AD (Accelerating Medicines Partnership-Alzheimer's Disease) project. There are seven cohorts in the dataset for parahippocampal gyrus.

Study	Brain Region	Cohort	Braak Stage								# Control (BRAAK 0-1)	#AD (BRAAK 5-6)	# Filtered Genes
Mount Sinai Brain Bank	Parahippocampal gyrus (BM36)	MSBB_BM36	0	1	2	3	4	5	6	Total	34	89	23201
			10	24	35	40	23	24	65	221			
