# Supplementary material for: Chromogranin A deficiency attenuates tauopathy by altering epinephrine–alpha-adrenergic receptor signaling in PS19 mice
Source: Nat Commun. 2025 May 20;16:4703. doi: 10.1038/s41467-025-59682-6 (PMC12092710; doi:10.1038/s41467-025-59682-6)
Supplement: Supplementary file 2 — Reporting Summary [file 41467_2025_59682_MOESM2_ESM.pdf]

nature portfolio

Corresponding author(s): Sushil K. Mahata, Gourisankar Ghosh

Last updated by author(s): Apr 19, 2025

Reporting Summary

Nature Portfolio wishes to improve the reproducibility of the work that we publish. This form provides structure for consistency and transparency in reporting. For further information on Nature Portfolio policies, see our Editorial Policies and the Editorial Policy Checklist.

Statistics

For all statistical analyses, confirm that the following items are present in the figure legend, table legend, main text, or Methods section.

n/a	Confirmed	
☐	☒	The exact sample size (*n*) for each experimental group/condition, given as a discrete number and unit of measurement
☐	☒	A statement on whether measurements were taken from distinct samples or whether the same sample was measured repeatedly
☐	☒	The statistical test(s) used AND whether they are one- or two-sided<br>*Only common tests should be described solely by name; describe more complex techniques in the Methods section.*
☒	☐	A description of all covariates tested
☐	☒	A description of any assumptions or corrections, such as tests of normality and adjustment for multiple comparisons
☐	☒	A full description of the statistical parameters including central tendency (e.g. means) or other basic estimates (e.g. regression coefficient) AND variation (e.g. standard deviation) or associated estimates of uncertainty (e.g. confidence intervals)
☐	☒	For null hypothesis testing, the test statistic (e.g. *F*, *t*, *r*) with confidence intervals, effect sizes, degrees of freedom and *P* value noted<br>*Give P values as exact values whenever suitable.*
☒	☐	For Bayesian analysis, information on the choice of priors and Markov chain Monte Carlo settings
☒	☐	For hierarchical and complex designs, identification of the appropriate level for tests and full reporting of outcomes
☐	☒	Estimates of effect sizes (e.g. Cohen's *d*, Pearson's *r*), indicating how they were calculated

Our web collection on statistics for biologists contains articles on many of the points above.

Software and code

Policy information about availability of computer code

Data collection	Keyence Fluorescence Microscope, UCSD Behavior core, UPLC, Biorad Chemidoc XR, Thermo Scientific TSQ 9610 GC-MS/MS, Novaseq S4, NIKON SoRa Spinning Disk Microscope
Data analysis	Excel v16.55, Prism v10.4.1, Image Lab, Image J, Thermo Chromeleon software, FASTQC, Trimmomatic (v0.39), STAR (v2.7.10b), StringTie (v1.3.6), prepDE.py3, DESeq2 (v1.42.0), ggplot2 (v3.4.4), ClusterProfiler (v4.10.0)

For manuscripts utilizing custom algorithms or software that are central to the research but not yet described in published literature, software must be made available to editors and reviewers. We strongly encourage code deposition in a community repository (e.g. GitHub). See the Nature Portfolio guidelines for submitting code & software for further information.

Data

Policy information about availability of data

All manuscripts must include a data availability statement. This statement should provide the following information, where applicable:

- Accession codes, unique identifiers, or web links for publicly available datasets
- A description of any restrictions on data availability
- For clinical datasets or third party data, please ensure that the statement adheres to our policy

RNAseq data was deposited in GEO (GSE274459). Mouse reference mm10 was used. Human data was provided.

Research involving human participants, their data, or biological material

Policy information about studies with human participants or human data. See also policy information about sex, gender (identity/presentation), and sexual orientation and race, ethnicity and racism.

Reporting on sex and gender	Sex was not considered in our study due to less sample size. Sex is mentioned in the table 1 and 2.
Reporting on race, ethnicity, or other socially relevant groupings	N/A
Population characteristics	N/A
Recruitment	Postmortem brain samples were obtained from UCSD ADRC (AD samples) and Mayo Clinic (CBD samples)
Ethics oversight	Since the samples were collected from post-mortem brain, this was exempted from UCSD Institutional Review Board.

Note that full information on the approval of the study protocol must also be provided in the manuscript.

Field-specific reporting

Please select the one below that is the best fit for your research. If you are not sure, read the appropriate sections before making your selection.

[x] Life sciences [ ] Behavioural & social sciences [ ] Ecological, evolutionary & environmental sciences

For a reference copy of the document with all sections, see nature.com/documents/nr-reporting-summary-flat.pdf

Life sciences study design

All studies must disclose on these points even when the disclosure is negative.

Sample size	We used G*Power 3.1 to determine the sample size for in vivo studies that provided 95% power to detect differences of 20-30% between experimental group means at alpha = 0.05 using ANOVA and Sidak's post hoc tests. Sample sizes and Statistical analyses for all experiments are included in the figure legends.
Data exclusions	We did not exclude any data.
Replication	For in vivo studies with mice, we performed more than 3 independent experiments using separate cohorts of animals to demonstrate reproducibility.
Randomization	Transgenic and knockout animals were randomized to treatment groups blindly by animal identifier and age to minimize bias and increase reproducibility.
Blinding	Biochemical assays were conducted in a blinded manner. All experimental groups will be coded and the code unblinded after the completion of the study.

Reporting for specific materials, systems and methods

We require information from authors about some types of materials, experimental systems and methods used in many studies. Here, indicate whether each material, system or method listed is relevant to your study. If you are not sure if a list item applies to your research, read the appropriate section before selecting a response.

Materials & experimental systems

n/a	Involved in the study	
[ ]	[x]	Antibodies
[ ]	[x]	Eukaryotic cell lines
[x]	[ ]	Palaeontology and archaeology
[ ]	[x]	Animals and other organisms
[ ]	[x]	Clinical data
[x]	[ ]	Dual use research of concern
[x]	[ ]	Plants

Methods

n/a	Involved in the study	
[x]	[ ]	ChIP-seq
[x]	[ ]	Flow cytometry
[x]	[ ]	MRI-based neuroimaging

Antibodies

Antibodies used	Immunohistochemistry:<br>AT8 (1:500), Thermo Fisher Science Cat# MN1020

	MC1 (1:500), Gift from P. Davies<br>CD68 (1:700), BIO-RAD Cat# MCA1957GA<br>GFAP (1:500), Cell Signaling Technology Cat# 3670<br>ADRA1B (1:500), Abcam, Cat# AB 169523<br>CgA (1:400) Invitrogen Cat# MA5-13093<br>NeuN (1:500) Proteintech 66836-1-Ig<br>MAP2 (1:400) Millipore-Sigma Cat#AB-5622<br>Anti-mouse Alexa Fluor 568 (1:300), Invitrogen Cat# A11031<br>Anti-rabbit Alexa Fluor 488 (1:300), Invitrogen Cat# A10042<br>Strep-Alexa Fluor 568 (1:500). Thermo Fisher Science Cat#S11226<br>Strep-Alexa Fluor 647 (1:500).Thermo Fisher Science Cat#S32357<br><br>Immunoblotting:<br>AT8 (1:3000), Thermo Fisher Science Cat# MN1020.<br>PHF1 (1:4000), Gift from P. Davies<br>CP13 (1:2000), Gift from P. Davies<br>HT7 (1:8000), Thermo Fisher Science Cat# MN1000<br>Actin (1:8000), Bio-Bharati, Cat# BB-AB0024<br>ADRA1B (1:2000), Abcam, Cat# AB 169523<br>ADRA2B (1:2000), Proteintech, Cat# 19778-1-AP<br>ADRB2 (1:2000), Cell Signaling Technology, Cat# 8513S<br>Alpha-Tubulin (1:6000), Cell Signaling Technology, Cat# 3873<br>CgA (1:2000) Invitrogen Cat# MA5-13093<br>Anti-Mouse-HRP (1:10000), Sigma, Cat# A0168<br>Anti-Rabbit-HRP (1:10000), Sigma, Cat# A0545<br>Strep-HRP (1:6000). Thermo Fisher Science Cat#N100
Validation	AT8, Thermo Fisher Science Cat# MN1020 (AT8 antibody has been used and cited in more than 200 publications and validated for immunoblotting and immunohistochemistry)<br>MC1, Gift from P. Davies (https://www.alzforum.org/alzantibodies/tau-mc-1)<br>CD68, BIO-RAD Cat# MCA1957GA (This antibody has been used in more than 200 publications and validated for IHC use)<br>GFAP, Cell Signaling Technology Cat# 3670 (GFAP antibody has been cited by more than 500 publications and validated for use in IHC)<br>ADRA1B, Abcam, Cat# AB 169523 (This antibody has been cited in more than 18 publications and validated for use in immunoblotting and immunohistochemistry)<br>CgA, Invitrogen Cat# MA5-13093. (Antibody has been cited by more than 20 publications and we have done immunohistochemistry where we did not get any signal from CgA-KO mice)<br>NeuN Proteintech 66836-1-Ig (This antibody has been used in more than 50 publications and is validated for IHC)<br>Anti-mouse Alexa Fluor 568, Invitrogen Cat# A11031 (Antibody has been used in more than 1000 publications and is being validated for immunohistochemistry)<br>Anti-rabbit Alexa Fluor 488, Invitrogen Cat# A10042 (Antibody has been used in more than 1000 publications and is being validated for immunohistochemistry)<br>Strep-Alexa Fluor 568. Thermo Fisher Science Cat#S11226 (This reagent has been cited more than 100 times and validated for use in immunohistochemistry)<br>PHF1, Gift from P. Davies (https://www.alzforum.org/alzantibodies/tau-phf-1)<br>CP13, Gift from P. Davies (https://www.alzforum.org/alzantibodies/tau-cp-13)<br>HT7, Thermo Fisher Science Cat# MN1000 (Antibody has been cited by more than 100 publications)<br>Actin, Bio-Bharati, Cat# BB-AB0024<br>ADRA2B, Proteintech, Cat# 19778-1-AP (The antibody is routinely tested by vendor for validation in immunoblotting)<br>ADRB2, Cell Signaling Technology, Cat# 8513S (The antibody is cited by 7 publications and validated by vendor for use in western blotting)<br>Alpha-Tubulin, Cell Signaling Technology, Cat# 3873 (The antibody is cited by more than 1000 publication and validated for use in western blotting)<br>Anti-Mouse-HRP, Sigma, Cat# A0168 (This antibody has been used for western blotting in more than 100 publications)<br>Anti-Rabbit-HRP (1:10000), Sigma, Cat# A0545 (This antibody has been used for western blotting in more than 100 publications)<br>Strep-HRP (1:6000). Thermo Fisher Science Cat#N100

Eukaryotic cell lines

Policy information about cell lines and Sex and Gender in Research

Cell line source(s)	ATCC #CRL-3275 (M. Diamond)
Authentication	https://www.atcc.org/products/crl-3275
Mycoplasma contamination	Negative
Commonly misidentified lines<br>(See ICLAC register)	*Name any commonly misidentified cell lines used in the study and provide a rationale for their use.*

Animals and other research organisms

Policy information about studies involving animals; ARRIVE guidelines recommended for reporting animal research, and Sex and Gender in Research

Laboratory animals	Chromogranin A knockout (CgA-KO) mice in B6 background were backcrossed to B6C3F1/J mice for 4 generations to generate CgA-KO mice in B6C3F1/J background. These mice were bred with hTau heterozygote mice from JAX (B6; C3-Tg(Prnp-MAPT*P301S) PS19Vle/J). CgA-Het/hTau-Het mice were bred with CgA-KO to generate CgA-KO/hTau mice. We used 4 different strains of mice: wild-type (B6C3F1/J), hTau {(B6; C3-Tg(Prnp-MAPT*P301S)PS19Vle/J}, CgA-KO, and CgA-KO/hTau mice.
Wild animals	None
Reporting on sex	We used both male and female mice. We were not powered to detect sex differences.
Field-collected samples	None
Ethics oversight	Experiments were performed with approval from the University of California San Diego Institutional Animal Care and Use Committee (Animal Welfare Assurance Number A3033-1 or D16-00020), which complies with the Animal Welfare Act, the Guide for the Care and Use of Laboratory Animals, the Public Health Service Policy on Humane Care and Use of Laboratory Animals, the US Government Principles for the Utilization and Care of Vertebrate Animals Used in Testing, Research, and Training, and the ARRIVE (Animal Research: Reporting In Vivo Experiments) guidelines for reporting animal experiments.

Note that full information on the approval of the study protocol must also be provided in the manuscript.

Clinical data

Policy information about clinical studies

All manuscripts should comply with the ICMJEguidelines for publication of clinical research and a completedCONSORT checklist must be included with all submissions.

Clinical trial registration	*Provide the trial registration number from ClinicalTrials.gov or an equivalent agency.*
Study protocol	*Note where the full trial protocol can be accessed OR if not available, explain why.*
Data collection	*Describe the settings and locales of data collection, noting the time periods of recruitment and data collection.*
Outcomes	*Describe how you pre-defined primary and secondary outcome measures and how you assessed these measures.*

Plants

Seed stocks	*Report on the source of all seed stocks or other plant material used. If applicable, state the seed stock centre and catalogue number. If plant specimens were collected from the field, describe the collection location, date and sampling procedures.*
Novel plant genotypes	*Describe the methods by which all novel plant genotypes were produced. This includes those generated by transgenic approaches, gene editing, chemical/radiation-based mutagenesis and hybridization. For transgenic lines, describe the transformation method, the number of independent lines analyzed and the generation upon which experiments were performed. For gene-edited lines, describe the editor used, the endogenous sequence targeted for editing, the targeting guide RNA sequence (if applicable) and how the editor was applied.*
Authentication	*Describe any authentication procedures for each seed stock used or novel genotype generated. Describe any experiments used to assess the effect of a mutation and, where applicable, how potential secondary effects (e.g. second site T-DNA insertions, mosiacism, off-target gene editing) were examined.*
